# Phenobarbital monotherapy for convulsive seizures in rural Northwest China: a 12-year longitudinal study

**DOI:** 10.1186/s42494-026-00252-8

**Published:** 2026-04-03

**Authors:** Xiaozhi Qiao, Yuanyuan Wang, Xiaomu Wang, Xiao Ma, Xuan Wang, Fang Yang, Changgeng Song, Lei Ma, Wen Jiang

**Affiliations:** Department of Neurology, The First Affiliated Hospital of Air Force Medical University, 127 Changle West Road, Xi’an, 710032 China

**Keywords:** Phenobarbital, Convulsive seizures, Rural area, Prognostic Factors

## Abstract

**Background:**

This study was conducted as part of the China Epilepsy Prevention and Control Management Project. The research aimed to investigate the efficacy of phenobarbital (PB) and prognostic factors for convulsive seizures in rural areas of northwest China.

**Methods:**

Patients with convulsive seizures were recruited from seven rural regions in Shaanxi Province, Northwest China, between January 2011 and December 2022. Patients over 6 years old and had at least one convulsive seizure at baseline (within 12 months prior to screening) were included. They were prescribed PB monotherapy according to a standardized protocol. During follow-up, the efficacy of PB was annually estimated by the reduction in seizure frequency. At the five-year follow-up, prognosis was assessed by the proportion of patients who achieved three consecutive seizure-free years.

**Results:**

A total of 1001 patients (mean age 40.9 years, 56.8% male) were included. The median follow-up duration was 5 years (interquartile range [IQR], 3–8; maximum 12 years). The median baseline seizure frequency was 5 seizures (IQR 3–12) per year. The proportion of patients achieving a 50% reduction in seizure frequency was 62.1% (622/1001) in the first year and 93.0% (133/143) by the tenth year, respectively. In total, 528 individuals achieved 3-year remission (3YR), 473 participants did not achieve 3YR. Patients who achieved 3YR demonstrated significantly higher rates of seizure freedom within the initial 24-month treatment period compared to non-3YR patients (89% vs 37%, respectively, *P* < 0.001). Multivariate analysis revealed that patients with a baseline seizure frequency exceeding 10 seizures per year had a significantly higher risk of failure to achieve 3YR (OR 2.13, 95%CI 1.63–2.78, *P* < 0.001). Adverse effects of PB were mostly mild, with 490 patients (48.9%) recording adverse reactions one month after prescription and decreased to 238 patients (23.8%) at their last visit (median follow-up 4 [IQR 2–7] years).

**Conclusions:**

Our study demonstrated that convulsive seizures could be effectively controlled with PB monotherapy with mild adverse effects in rural areas in Northwest China. Early treatment response, particularly seizure freedom within the first two years, is a strong indicator of long-term remission. High baseline seizure frequency is an independent risk factor for poor prognosis.

**Supplementary Information:**

The online version contains supplementary material available at 10.1186/s42494-026-00252-8.

## Background

The lifetime prevalence of epilepsy was approximately 7‰ worldwide, and it was estimated that around 10 million people with epilepsy (PWE) reside in China [1, 2]. PWE generally experience high rates of disability and mortality [3]. In China the age-standardized disability-adjusted life years (DALYs) due to epilepsy was 99.77 per 100,000 population, imposing a heavy burden on PWE, caregivers and the health-care systems [4]. Convulsive seizures are the most harmful seizures and associated with sudden unexpected death in epilepsy [5]. In rural China, the risk of premature death among patients with convulsive seizures is approximately three times higher than that of the general population [6].

Generally, seizures could be controlled in approximately 70% of PWE through the regular use of anti-seizure medications (ASMs) [7, 8]. However, an ILAE/IBE/WHO study in 2003 showed that, around two-thirds of people with active epilepsy in China did not receive appropriate treatment [1]. This treatment gap was largely attributed to misconceptions towards epilepsy, a lack of relevant knowledge, limited availability of ASMs and insufficient neurological expertise required for the diagnosis and management of epilepsy [9, 10].

To narrow the treatment gap, the World Health Organization (WHO) in cooperation with the International League Against Epilepsy (ILAE) and the International Bureau for Epilepsy (IBE), launched the Global Campaign Against Epilepsy (GCAE) in 1997 [11]. As part of this Campaign, China has established the Demonstration Project “Epilepsy Management at a Primary Health Level” (EMPHL) since 2000 [12, 13]. This project developed a PB-based intervention protocol, primarily implemented by primary healthcare providers, to manage convulsive seizures (generalized tonic–clonic seizures [GTCS] or secondarily GTCS [sGTCS]) in rural settings. The standardized protocol has proven effective in reducing seizure frequency with mild adverse events and has contributed to narrowing the epilepsy treatment gap [14, 15]. In 2005, the Epilepsy Prevention and Control Management Project (EPCMP) was launched as an extension of the demonstration project to more rural areas of China [16].

Since 2011, seven counties and districts of Shaanxi province have participated in the EPCMP, covering approximately 2.55 million residents. This study aimed to evaluate the prognosis of PB monotherapy for convulsive seizures in rural areas in Shaanxi province, Northwest China.

## Materials and methods

### Study population

This study was conducted in seven counties and districts (Sanyuan County, Huayin County, Huangling County, Nanzheng County, Fengxiang District, Hanbin District, Huyi District) in the rural areas of Shaanxi province. The study area encompassed over 100 towns, and approximately 2.55 million residents. Primary healthcare services were delivered through approximately 130 village and town healthcare stations. Patients were recruited between January 2011 and December 2022. Written informed consent was obtained from all participants or their guardians (patient under the age of 16).

### Procedures and participants

Prior to the initiation of the study, all primary-care workers received basic training from neurologists in the diagnosis and management of convulsive seizures, as well as in the use of the screening questionnaire and follow-up forms.

The details of the operating procedures had been described in previous publications related to the demonstration project [14]. In brief, patients were defined as having convulsive seizures (GTCS or sGTCS) if they exhibited two of the first three following symptoms and any one of the last three symptoms: (1) loss of consciousness; (2) rigidity; (3) generalized convulsive movements; (4) urinary incontinence; (5) a bitten tongue or an injury from a fall; (6) post-seizure fatigue, drowsiness, headache or muscle aches. The diagnosis of convulsive seizures was subsequently confirmed by senior neurologists. Patients aged over 6 years who had experienced at least one convulsive seizure within the 12 months prior to screening (baseline) and who had either never received treatment or had been on irregular treatment with ASMs in the month prior to screening were eligible for inclusion into the study.

Patients were excluded if they met any of the following criteria: (1) provoked seizures only (e.g., seizures associated with pregnancy, alcohol withdrawal or drug withdrawal); (2) age < 6 years or weight < 10 kg; (3) a history of progressive neurological diseases; (4) a history of status epilepticus; (5) the presence of cardiac, hepatic, or renal disorders, or severe hypertension; (6) allergy to PB.

The project office monitored the implementation of the study, conducting training, supervision, and assessment at regular intervals.

### Data collection and follow-up

Baseline data were collected during the screening process, including age at epilepsy onset, duration of epilepsy, frequency of convulsive seizures, history of ASMs treatment. Follow-up surveys were conducted by local primary healthcare workers every two weeks for the first two months, and then conducted monthly door-to-door. These healthcare workers were responsible for drug delivery and filling out follow-up forms, which recorded medication adherence, prescribed PB dosage, the number of convulsive seizures, and adverse events at each visit. For patients aged ≥ 15 years or with a body weight ≥ 30 kg, the initial PB dosage was 60 mg per day, administered at bedtime. This dosage was increased to 90 mg after two weeks if patients experienced more than one seizure. The maximum dosage did not exceed 210 mg per day. For patients aged < 15 years or with a body weight < 30 kg, the initial dosage was 2 mg/kg/day, which could be increased to 3 mg/kg/day if seizures remained uncontrolled. The maximum dosage typically did not exceed 5 mg/kg/day.

Participants in our cohort were followed until December 2023 when data collection was terminated. Discontinued patients include those who withdrew, were lost to contact, died, changed ASM regimens or did not complete follow-up due to late enrollment. Patients were required to withdraw from treatment if they met one or more of the following conditions: (1) an allergic reaction (rash) caused by PB; (2) opposition to continued treatment by the patient or guardian; (3) non-compliance with medical advice; (4) the development of a progressive disorder of nervous system, heart, liver or kidneys; (5) intolerable adverse effects of PB; (6) being lost to follow-up for more than six months within any 12-month period; or (7) no reduction or an increase in seizure frequency despite full-dose guideline-compliant therapy.

### Efficacy and adverse effects assessment

The efficacy of PB was evaluated annually after treatment initiation, based on the reduction in convulsive seizure frequency over the past 12 months compared to the baseline frequency (within 12 months before screening). Efficacy at each observation point was classified as follows: (1) seizure-free: no seizure during the past 12 months; (2) effective: ≥ 50% reduction in seizure frequency; (3) ineffective: < 50% reduction in seizure frequency; and (4) deteriorated: an increase in seizure frequency.

Seizure freedom during follow-up was categorized into 1-year remission (1YR), 3-year remission (3YR) and 5-year remission (5YR), defined as the completion of 1, 3, or 5 consecutive seizure-free years, respectively. Relapse was defined as the recurrence of seizures after achieving remission for one year or more. Prognosis was assessed based on the proportion of patients entering 3YR within the 5-year follow-up (representing over 50% of the study population). Poor prognosis was defined as failure to achieve 3YR by the 5-year follow-up.

The adverse effects of PB were assessed by trained primary-care workers using a questionnaire. The severity of symptoms, including exhaustion, drowsiness, ataxia, dizziness, headache, hyperactivity, skin rash, gastrointestinal symptoms, and emotional disturbance (including depression and anxiety), was rated on a scale of none, mild, moderate, or severe.

In cases of mild to moderate adverse reactions, dose escalation should be temporarily withheld while maintaining close clinical monitoring. For severe adverse reactions, PB should be discontinued immediately, with appropriate diagnostic workup initiated to evaluate and monitor for potential complications. Importantly, any allergic reaction (including rash) necessitates prompt discontinuation of PB regardless of severity, followed by substitution with an alternative ASM.

### Statistical analysis

All data were organized using EpiData software (The EpiData Association, Odense, Denmark). Data analysis was performed using SPSS 22.0 for Windows (IBM Corp., Armonk, NY, USA). Data were presented as numbers with percentages, means with standard deviations (SD), or medians with interquartile ranges (IQR), as appropriate. Differences between categorical variables were assessed using the Pearson chi-square test. Numeric variables were compared between groups using one-way ANOVA for normally distributed data and Kruskal–Wallis test for non- normally distributed data.

To assess attrition bias, the baseline characteristics of participants who completed the 3YR evaluation at the 5-year follow-up were compared with those who did not. Multiple imputation with chained equations (MICE), incorporating 20 imputed datasets, was employed to handle missing seizure outcomes.

Logistic regression was applied to investigate the risk factors for poor prognosis. All variables were initially evaluated for significance using univariate analysis with a *P*-value < 0.15 as the threshold for inclusion in the multivariable logistic regression model. Variables exhibiting significant collinearity were excluded. A *P*-value < 0.05 was considered statistically significant. As sensitivity analysis, the multivariable logistic regression model for poor prognosis was repeated without imputation of missing data.

## Results

### Patient demographics

**Fig. 1:**
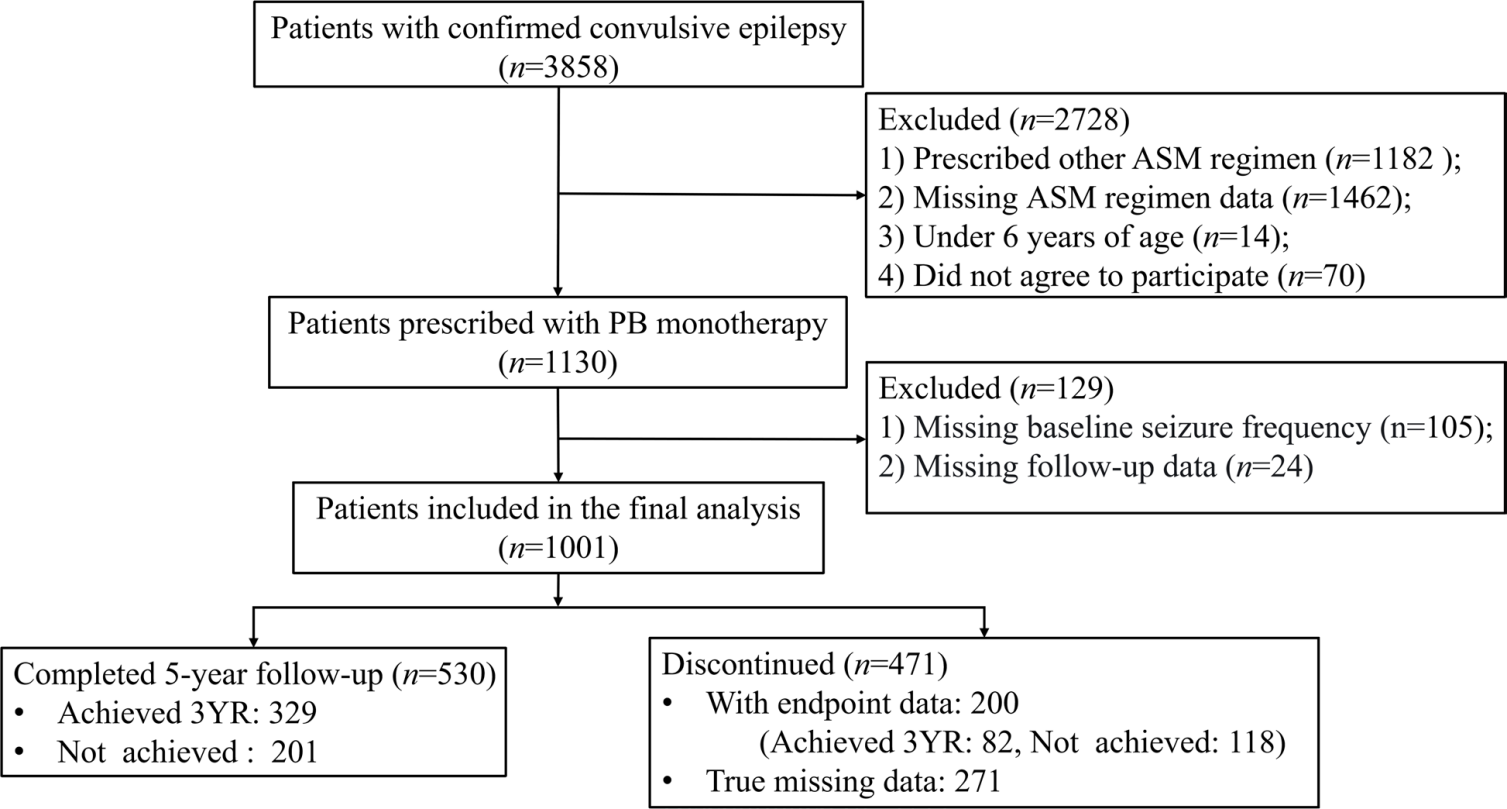
Flow chart describing the study population. ASMs = anti-seizure medications

A total of 1001 individuals were included in the study. Among them, 530 participants (52.9%) completed the 5-year follow-up, including 329 who achieved 3YR and 201 who did not. Treatment discontinuation occurred in 471 patients (47.1%), of whom, 200 had evaluable endpoint data prior to dropout (82 achieving 3YR and 118 not achieving). The remaining 271 patients were classified as true missing data due to dropout before reaching the predefined endpoint assessment window (Fig. 1).

Table 1 summarizes basic demographic and seizure-related parameters of the whole study cohort. The cohort comprised 569 males (56.8%). The median follow-up duration was 5 years (IQR 3–8). The median baseline seizure frequency was 5 seizures per year (IQR 3–12) within the 12 months prior to screening. Among them, 579 of 1001 (57.8%) had not taken any ASM within 1 year before enrollment, including 195 had never received treatment.

**Table 1:** Patient demographics

	**Total** **(n = 1001)**	**Completed (n = 730)**	**Discontinued** **(n = 271)**	**Completed Vs. discontinued** ***P* value**
Age, mean (SD), y	40.93 (17.05)	40.22 (16.78)	42.82 (17.65)	0.032*
Sex, male, *n* (%)	596 (56.8%)	418 (57.26%)	151 (55.72%)	0.662
BMI, mean (SD)	21.87 (3.16)	21.85 (3.17)	21.91 (3.13)	0.791
Age at epilepsy onset, Mid (IQR), y	18 (10–31.5)	17 (9–30)	20 (10–34)	0.162
Duration of epilepsy, Mid (IQR), y	16 (7–27)	15.5 (8–26)	16 (6–27)	0.868
Baseline seizure frequency, Mid (IQR)	5 (3–12)	5 (3–12)	6 (3–15)	0.197
ASMs treatment within 1 year before enrollment, *n* (%)	422 (44.2%)	327 (44.79%)	115 (42.43%)	0.504
History of ASMs treatment, *n* (%)	806 (80.5%)	584 (80%)	222 (81.92%)	0.496

Baseline seizure frequency: the number of convulsive seizures within one year before the screening

*Abbreviations*: *ASMs *anti-seizure medications, *BMI *Body mass index, *IQR *interquartile range, *Mid *median, *SD *standard deviation, *n *numbers, *y *years

^*^Data in bold and marked with ^*^ indicate statistical significance (*P *< 0.05)

In addition, subsets of patients completed the 3YR assessment within 5 years (*n* = 730) and those who discontinued from the study (*n* = 271) were compared (Table 1). Discontinuation reasons (*n* = 271) included: 46 withdrew consent (39 due to migration), 41 lost to contact, 2 adverse events, 129 exclusions (81 due to changes in ASMs, 13 poor compliance, 35 missing follow-up records), 29 as a result of study closure, and 24 deaths. Patients who discontinued from the study were significantly older than those who completed the follow-up (mean difference 2.6 years, *P* = 0.036). However, there were no significant differences in key baseline characteristics including BMI, age at epilepsy onset, duration of epilepsy, baseline seizure frequency and history of ASMs treatment (all *P* > 0.05) (Table 3).

### Efficacy and prognosis of PB monotherapy

**Fig. 2:**
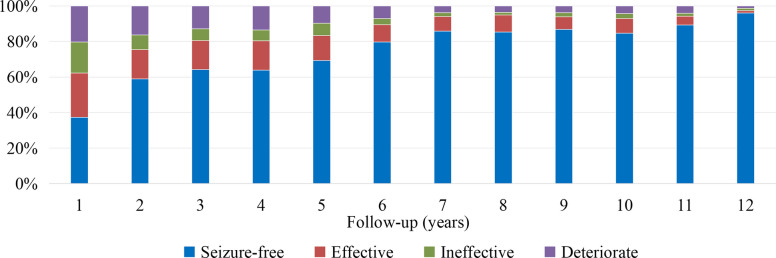
The efficacy of phenobarbital monotherapy treatment during 12-year follow-up

The retention rate of participants in the study was 76.0% (761/1001) at the 3-year visit, 52.9% (530/1001) at the 5-year visit, and 14.3% (143/1001) at the 10-year visit. The efficacy was assessed based on the reduction in convulsive seizure frequency compared to baseline. A reduction of over 50% in seizure frequency was observed in 622 of 1001 (62.1%) participants at the first year, 612 of 761 (80.4%) at the third year, 442 of 530 (83.4%) at the fifth year, and 133 of 143 (93.0%) at the tenth year, respectively (Fig. 2). The percentage of participants who achieved seizure-free was 37.2% at the first year, 64.1% at the third year, and 84.6% at the tenth year, respectively (Fig. 2).

**Fig. 3:**
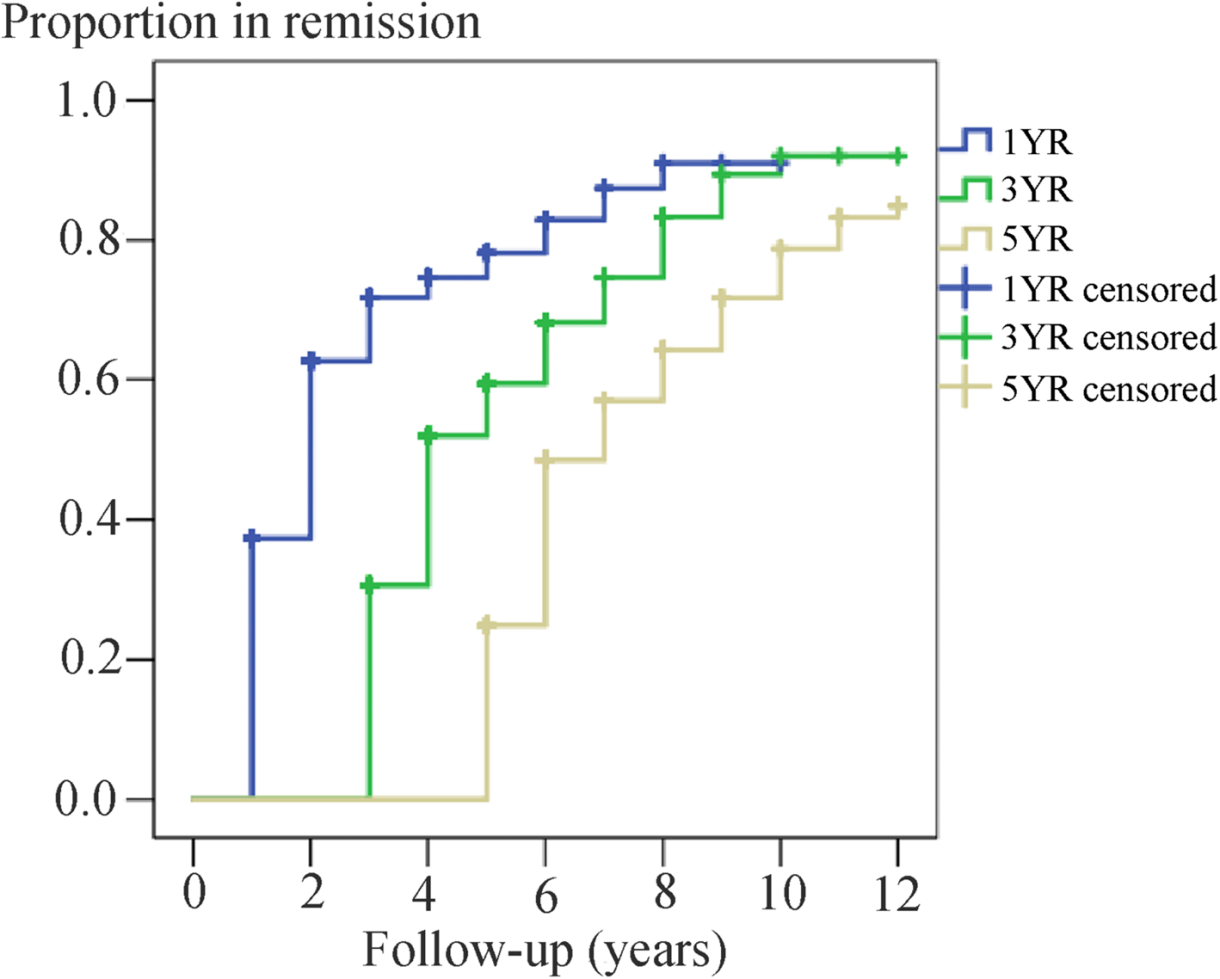
Kaplan–Meier estimates of the cumulative probability of 1YR, 3YR and 5YR. 1YR = 1-year remission; 3YR = 3-year remission; 5YR = 5-year remission

**Fig. 4:**
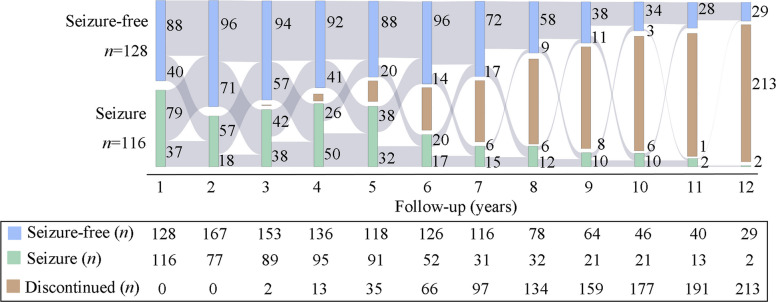
The remission-relapse pattern (*n* = 244) during 12 years of follow-up was displayed by Sankey diagram. Discontinued is composed of patients who withdrew, were lost to contact, dead, changed anti-seizure medicine regimens or did not complete follow-up due to late enrollment

During the follow-up, 69.1% (692/1001) of the participants could achieve one or more 1YR. A total of 475 participants achieved 3YR with a median follow-up duration of 7 years (IQR 5–11), and 293 participants achieved 5YR with a median follow-up duration of 9 years (IQR 7–11). The cumulative probabilities of achieving 1YR, 3YR and 5YR were estimated to be 0.91, 0.92 and 0.83, respectively at 10 years after treatment initiation, as calculated by the Kaplan–Meier survival curve (Fig. 3). At the last follow-up, 448 patients had not relapsed since their first 1YR, while 244 patients experienced a remission-relapse pattern (Fig. 4).

Additionally, 309 patients never achieved seizure-free with a median follow-up of 2 years (IQR 1–4). At the final visit, treatment was effective for 41.7% of patients, ineffective for 20.7%, and deteriorate in 37.5%. Most of them were then subsequently changed medication regimens (*n* = 89, 28.8%), lost to contact (*n* = 79, 25.6%), or withdrawn from the study (*n* = 53, 17.2%).

Complaints on adverse effects were occurred to 48.95% (490/1001) of patients within the first month after prescription (Table 2). Major adverse effects of PB included mild exhaustion, drowsiness, dizziness, headache and gastrointestinal symptoms. Only 30 of 1001 (3.0%) patients experienced moderate to severe adverse effects. The incidence of adverse effects decreased with longer follow-up period. At the last visit, only 23.8% (238/1001) of the patients reported mild to moderate adverse events.

**Table 2:** Complain of adverse effects during initial phenobarbital monotherapy (first month)

	**Exhaustion/** **Drowsiness**	**Dizziness**	**Headache**	**Gastrointestinal symptoms**	**Depression, anxiety**	**Ataxia**	**Hyperactivity**	**Skin rash**
Mild	393 (39.3%)	282 (28.2%)	169 (16.9%)	156 (15.6%)	104 (10.4%)	92 (9.2%)	42 (4.2%)	33 (3.3%)
Moderate to severe	15 (1.5%)	13 (1.3%)	8 (1%)	3 (0.3%)	7 (0.7%)	2 (0.2%)	5 (0.5%)	4 (0.5%)
Total	408 (40.8%)	295 (29.5%)	177 (17.7%)	159 (15.9)	111(11.1%)	94 (9.4%)	47 (4.7%)	38 (3.8%)

### Risk factors of poor prognosis

Following MICE to address missing data, our analysis included 1,001 participants, of whom 528 (52.7%) achieved 3YR within 5-year follow-up while 473 (47.3%) did not. The 3YR group demonstrated significantly earlier treatment response, with 89% attaining the first 1YR within two years compared to only 37% of non-3YR patients achieving the first 1YR during this period (*P* < 0.001) (Table S1). These findings highlight that early remission is an indicator of long-term remission.

Univariate analysis identified two factors were associated with poor prognosis: baseline seizure frequency (*P* < 0.001), and treatment history (*P* < 0.05). Multivariate analysis showed that higher baseline seizure frequency (OR 2.13, 95%CI 1.63–2.78, *P* < 0.001) was significantly associated with an increased failure to achieve 3YR (Table 3).

**Table 3:** Logistic regression analysis the risk factors related to poor prognosis with phenobarbital monotherapy

	**3YR** **(n = 518)**	**No 3YR** **(n = 483)**	**Univariable analysis**			**Multivariable analysis**		
Variables	**N (%)**	**N (%)**	**OR**	**95%CI**	***P*-value**	**OR**	**95%CI**	***P*-value**
Female sex	528 (43.4)	203 (42.9)	0.98	0.76–1.26	0.885			
BMI					0.340			
18.5–23.9	341 (64.6)	322 (68.1)						
< 18.5	63 (11.9)	58 (12.3)	0.98	0.66–1.44	0.898			
≥ 24	124 (23.5)	93 (19.7)	0.79	0.59–1.08	0.144			
Age at epilepsy onset (y)					0.676			
0–5	79 (15.0)	76 (16.1)						
6–17	172 (32.6)	167 (35.3)	1.01	0.69–1.48	0.962			
18–59	250 (47.3)	209 (44.2)	0.87	0.60–1.25	0.450			
≥ 60	27 (5.1)	21 (4.4)	0.81	0.42–1.55	0.522			
Duration (y)					0.875			
< 10	161 (30.5)	154 (32.6)						
< 20	140 (26.5)	127 (26.8)	0.95	0.68–1.31	0.750			
< 30	112 (21.2)	95 (20.1)	0.89	0.62–1.26	0.503			
≥ 30	115 (21.8)	97 (20.5)	0.88	0.62–1.25	0.480			
Baseline seizure frequency ≥ 10 per year	145 (27.5)	217 (45.9)	2.24	1.72–2.91	***P* < 0.001**	2.13	1.63–2.78	*P* < 0.001*
Treatment history	409 (77.5)	397 (83.9)	1.52	1.11–2.09	**0.010**	1.29	0.93–1.79	0.128
PB dosage ≥ 90 mg/day	211 (40.0)	196 (41.4)	1.06	0.83–1.37	0.635			
Having adverse effect	273 (51.7)	218 (46.1)	0.80	0.62–1.02	**0.076**	0.89	0.69–1.16	0.392

PB dosage referred to the dose taken by the patient at the last visit

*P* < 0.15 in univariable analysis are bolded

*3YR *3-year remission, *N *number of patients, *OR *Odds ratios, *CI *confidence interval, *BMI *body mass index, *y *years

^*^Was significantly (*P* < 0.05) associated with 3YR in multivariable analysis

The sensitivity analysis confirmed the same predictor when computing a logistic regression model without imputations (OR 2.27, 95%CI 1.65–3.12, *P* < 0.001) (Table S2).

## Discussion

Our data demonstrated that PB monotherapy yielded effective outcomes in 62.1% (622/1001) of patients at 1 year and 93.0% (133/143) at 10 years. During the follow-up, 692 of 1001 patients (69.1%) achieved remission for one year or longer, including 475 patients entered 3YR, and 293 patients entered 5YR. Among these, 448 patients did not relapse after their first 1YR, while 244 patients experienced a remission-relapse pattern. Early remission, particularly within the first two years of treatment, was a strong indicator of long-term remission outcome. Additionally, patients with a baseline seizure frequency exceeding 10 seizures per year carried a 2.1-fold risk (OR 2.13, 95%CI 1.63–2.78, *P* < 0.001) of poor prognosis.

Although novel ASMs have been introduced into clinical practice, the overall rate of seizure control has not significantly improved [7]. PB is economical and is still recommended as a first-line therapy in resource-poor regions by the WHO [17]. The advantages of PB include its effectiveness against a broad spectrum of seizure types, once-daily dosing, a low risk of life-threatening adverse effects, and low cost (around 3 USD per person per year) [18]. Adverse reactions reported in rural areas of China indicated that PB was generally well-tolerated. Despite the use of high doses, serious adverse effects were uncommon [19, 20]. In our study, most patients experienced no adverse effects or only mild adverse effects, such as mild exhaustion, drowsiness, dizziness, headache and gastrointestinal symptoms. Moderate to severe adverse effects were rare. These findings further support the feasibility of PB for epilepsy management in low- and middle-income countries.

The efficacy of PB monotherapy in rural areas of China had been previously described [21, 22]. After 12 months of treatment, seizure frequency was reduced by at least 50% in 68−81.3% of patients [14, 21, 23]. Our results showed similar efficacy, showing that 62.1% of patients achieved a ≥ 50% reduction in seizure frequency during the first year, with 37% achieving complete seizure freedom. A recent study evaluated the efficacy of PB monotherapy in adult women in rural China, reporting that 89.4% of patients were seizure-free after seven years of treatment, increasing to 96.6% by the 10th year [24]. Our study, which assessed efficacy at 12-month intervals, further demonstrated that the treatment effectiveness improved with longer follow-up durations. This improvement may be attributed to: (1) sustained treatment leading to better seizure control; (2) patients with poor efficacy were more likely to withdraw from the study over time.

During follow-up, 244 patients experienced a remission-relapse pattern, which was speculated to be related to poor medication compliance, underlying etiologies of epilepsy, or specific triggers such as fatigue, illness, dietary factors. [25, 26]. Alternating remission-relapse might increase the risk of developing refractory epilepsy [27]. These patients required more intensive management, including detailed diagnostic evaluations to identify and address the underlying causes of their epilepsy.

The ultimate goal of epilepsy treatment is the complete withdrawal of ASMs without relapse. The reported factors with prognostic value for seizure outcomes include etiology, electroencephalogram (EEG) abnormalities, GTCS, and the number of seizures before and after treatment initiation [28–30]. A population-based cohort study of adults with newly diagnosed epilepsy showed that seizures occurring within one year of starting ASMs treatment were predictive of refractory epilepsy [31]. These findings were in line with our data, which indicated that poor prognosis was associated with higher baseline seizure frequency and inadequate response to PB treatment in the initial two years.

### Limitations

Several limitations of this study should be pointed out. First, self-reported information may introduce recall bias. Second, the progressive increase in dropout rates during follow-up, coupled with incomplete documentation of withdrawal reasons, may introduce selection bias that could compromise the validity of the effectiveness outcomes. Although MICE was employed, residual bias may persist due to unmeasured confounding factors influencing dropout (e.g., unreported adverse effects or insufficient treatment efficacy). These limitations warrant cautious interpretation of the treatment outcomes. Third, adverse reaction assessment relied on self-scaling without therapeutic drug monitoring, laboratory or formulated scales. While good tolerability was observed at low PB doses, this methodological limitation may affect data reliability. Additionally, pregnancy-related management and side effects were not assessed due to constraints in the initial study design. Future large-scale studies are needed to establish PB safety in pregnant women lacking access to alternative antiepileptic therapies. Fourth, due to constrained diagnostic resources in rural settings, the diagnosis of convulsive seizures (GTCS or sGTCS) was primarily based on clinical presentation. This pragmatic approach, while necessary under the circumstances, may introduce heterogeneity in treatment responses. Last, most patients enrolled in the study did not undergo video-EEG or MRI examinations, which might be a valuable prognostic factor for long-term seizure outcome. These limitations highlight the need for: (1) enhanced healthcare worker training and patient education to improve management; (2) implementation of objective monitoring tools; and (3) diagnostic capacity building in underserved areas to optimize epilepsy management.

## Conclusions

Our study demonstrated that the convulsive seizures of patients with epilepsy could be effectively controlled by PB monotherapy with tolerable adverse effects in rural areas in Northwest China. This result further supports the recommendation of phenobarbital as a first-line therapy in resource-poor areas. Furthermore, baseline seizure frequency and the efficacy of PB monotherapy within the first 2 years of treatment had significant prognostic value in rural settings. This study provides new data for primary healthcare workers to better manage convulsive seizures and holds potentials for improving patients' quality of life.

## Supplementary Information

Supplementary Material 1

## Data Availability

The data that support the findings of this study are available from the corresponding author upon reasonable request.

## Abbreviations

ASMs: Anti-seizure medications
BMI: Body Mass Index
DALYs: Disability-adjusted life years
EEG: Electroencephalogram
EPCMP: Epilepsy Prevention and Control Management Project
GTCS: Generalized tonic–clonic seizures
LOCF: Last observation carried forward
PB: Phenobarbital
PWE: People with epilepsy
sGTCS: Secondarily generalized tonic–clonic seizures
1YR: One-year remission
3YR: Three-year remission
5YR: Five-year remission
