# Supplementary material for: Phenobarbital monotherapy for convulsive seizures in rural Northwest China: a 12-year longitudinal study
Source: Acta Epileptol. 2026 Apr 3;8:14. doi: 10.1186/s42494-026-00252-8 (PMC13047833; doi:10.1186/s42494-026-00252-8)
Supplement: Supplementary file 1 — Supplementary Material 1 [file 42494_2026_252_MOESM1_ESM.doc]

Supplementary table 1. Association between early remission and 3-year remission (3YR) status

|  | **3YR (n=528)** | **non-3YR (n=473)** | ***P*-value** | **Effect size (Cramer's V)** |
| --- | --- | --- | --- | --- |
| **Early remission** | 470 (89%) | 175 (37%) | *P*<0.001* | 0.54 |
| **Late remission** | 58 (11%) | 298 (63%) |

* Statistically significant differences (*p*<0.05)

Supplementary table 2. Prognostic factors for poor outcome according to multivariable logistic regression analysis without imputing missing data (complete case analysis)

|  | **3YR**  **(n=411)** | **No 3YR**  **(n=319)** | **Multivariable analysis** | | |
| --- | --- | --- | --- | --- | --- |
| **Variables** | **N (%)** | **N (%)** | **OR** | **95%CI** | ***P*-value** |
| **Baseline seizure frequency ≥10 per year** | 104 (25.3) | 142 (44.5) | 2.27 | 1.65-3.12 | ***P*<0.001*** |
| **Treatment history** | 315 (76.6) | 269 (84.3) | 1.41 | 0.95-2.07 | 0.086 |
| **Having adverse effect** | 182 (44.3) | 148 (46.4) | 1.00 | 0.74-1.35 | 0.999 |

BMI= body mass index; OR= Odds ratios; *Was significantly (*p* < 0.05) associated with 3-year remission in multivariable analysis.
